# Bibliographical

**Published:** 1867-06

**Authors:** 


### BIBLIOGRAPHICAL.

*Pereira's Materia Medica and Therapeutics Edited, with numerous references to the United States Pharmacopœia, and many other additions*, by Horatio C. Wood, Jr., M. D. Published by Henry C. Lea, Philadelphia: 1866.

In this work we have an abridged edition, adapted to the use of students and physicians in America. While many things contained in the English editions are left out, additions comprising subjects more useful to us are made. In these alterations, however, we have the work reduced to the size and compromising those items which makes it suitable as a text-book for the student.

While the classification of remedies is not such in our opinion is best suited to the Student of Medicine, we know of no recent work on Materia Medica, to which the same objection does not obtain.

We are great admirers of the talent and research of the great English teacher, and hail with pleasure the appearance of his great work in a form to make it suited to the American Student of Medicine.

We are also in receipt of the fifth American, from the fourth London edition of Headland, on the action of medicine.

The subject of this work is an important one, and the manner in which it is treated makes the volume an important addition to the library of every physician.

The manner in which certain changes are produced in the animal organism, by remedial agents, is so important to the understanding of rational medicine, that the absence of such knowledge leaves the practitioner without the pale of scientific medicine.

We commend the book to those wishing a work on this subject.

It is published by Lindsay and Blakiston, Philadelphia: 1867.

We have before us the fifth edition revised, of the Art and Science of Obstetrics. By Charles D. Meigs, M. D., &c. Henry C. Lea, Philadelphia: 1867.

